# Recyclable Magnetic Titania Nanocomposite from Ilmenite with Enhanced Photocatalytic Activity

**DOI:** 10.3390/molecules22122044

**Published:** 2017-11-23

**Authors:** Tianjie Hong, Jun Mao, Feifei Tao, Mingxuan Lan

**Affiliations:** 1 Department of Chemistry and Chemical Engineering, Shaoxing Univeristy, Shaoxing 312000, China; hongtianjie001@163.com (T.H.); maojune666@163.com (J.M.); 13467181280@163.com (M.L.); 2 Shanghai Advanced Research Institute, Chinese Academy of Sciences, Shanghai 201210, China

**Keywords:** ilmenite, titania, nanocomposite, photocatalysis, magnetic recyclability

## Abstract

Using ilmenite as a raw material, iron was converted into Fe_3_O_4_ magnetic fluid, which further was combined with titanium filtrate by a solvothermal method. Finally Fe_3_O_4_/TiO_2_ nanocomposites with the uniform size of 100–200 nm were prepared. This approach uses rich, inexpensive ilmenite as a titanium and iron source, which effectively reduces the production cost. The crystal structure, chemical properties and morphologies of the products were characterized by SEM, TEM, XRD, FTIR, BET, UV-Vis, XPS and VSM. The novel photocatalyst composed of face-centered cubic Fe_3_O_4_ and body-centered tetragonal anatase–TiO_2_ exhibits a spherical shape with porous structures, superparamagnetic behavior and strong absorption in the visible light range. Using the degradation reaction of Rhodamine B (RhB) to evaluate the photocatalytic performance, the results suggest that Fe_3_O_4_/TiO_2_ nanocomposites exhibit excellent photocatalytic activities and stability under visible light and solar light. Moreover, the magnetic titania nanocomposites displayed good magnetic response and were recoverable over several cycles. Based on the trapping experiments, the main active species in the photocatalytic reaction were confirmed and the possible photocatalytic mechanism of RhB with magnetic titania was proposed. The enhanced photocatalytic activity and stability, combined with excellent magnetic recoverability, make the prepared nanocomposite a potential candidate in wastewater purification.

## 1. Introduction

With the rapid development of human society, environmental pollution and energy shortages have become the two major problems which need more attention to. The use of photocatalysis technology, applying solar energy efficiently to achieve photocatalytic degradation of organic pollutants [1,2,3,4], is an effective way to solve the above stated problems. Among various photocatalysts, due to superior catalytic activity, chemical inertness, nontoxicity and low cost, titanium dioxide (TiO_2_, titania) has become the most popular material in photocatalytic research and application [5,6,7,8]. However, pure TiO_2_ can be activated only by ultraviolet light which is less than 5% of the total solar energy reaching earth due to its wide band gap (3.0 to ~3.2 eV) [9], and exhibits a low quantum efficiency due to the easy recombination of photogenerated electrons and holes [10]. In addition, in order to improve the photocatalytic activity of TiO_2_, nanostructured TiO_2_ is often expected to exhibit outstanding photocatalytic performance, owing to their larger specific surface area and good dispersion in aqueous solutions [11]. However, in practical treatment processes, finely dispersed TiO_2_ particles are difficult to separate and recover from a liquid phase [12]. Therefore, how to separate and recover nano-TiO_2_, and improve the visible-light-driven nano-TiO_2_, have been research hotspots [13,14].

Recently, magnetic titania has gained increasing attention, as it has the advantages of magnetic recovery and excellent photocatalytic properties [15,16,17,18,19]. They can achieve the fast separation and easy recovery of nano-TiO_2_ by external magnetic fields, thus preventing the loss of active components and improving the durability of catalysts. Also magnetic titania can reduce the cost incurred by ultrafiltration or centrifugation, which is used to recover the power photocatalyst [20]. Fe_3_O_4_, as a ferrite magnetic material, has strong magnetic properties and can be used as a carrier to prepare magnetic TiO_2_ photocatalyst that can be effectively separated and recovered with the help of an external magnetic field [21,22]. S. Khashan et al. used a modified sol-gel method to prepare Fe_3_O_4_@TiO_2_ core/shell nanoparticles, which exhibited excellent magnetic separation and targeting [21]. W.-F. Jiang et al. combined sol-gel and simple hydrothermal methods to construct a magnetic composite of Fe_3_O_4_@TiO_2_ core-shell microspheres supported by silica aerogels, which possessed good photocatalytic activity and excellent magnetic properties, enhanced charge separation efficiency and strong light absorption in the visible region [22]. Meanwhile, the Fe_3_O_4_/TiO_2_ composite can reduce the band gap of TiO_2_ and extend the adsorption wavelength range to the visible region, which further improves the photocatalytic activities [23]. Moreover, almost all of the titanium sources used are expensive organic or inorganic raw materials, and the procedure for preparing magnetic titania often requires rigorous reaction conditions, hence limiting its application. Also the magnetic properties are often influenced by a multilayer coating and high temperature calcinations, thus affecting the photocatalytic efficiency [24]. Therefore a facile approach to the low cost preparation of the magnetic titania nanocomposite at relatively low temperatures is highly desired.

As the major mineral source of titania across the world, ilmenite (FeTiO_3_) is cheap and contains titanium and iron. The fabrication of magnetic TiO_2_ nanocomposite directly from ilmenite can greatly reduce production costs, and it shows potential for application in large scale industry. In view of this, titanium and iron were first extracted directly from ilmenite, and then Fe_3_O_4_ magnetic fluid was prepared from the iron extraction and taken as a carrier to combine with the extracted titanium precursor. Finally, the magnetic titania nanocomposite (Fe_3_O_4_/TiO_2_) was synthesized by a solvothermal method that doesn’t require high temperature annealing. Fe_3_O_4_ magnetic fluid, used as the magnetic core, has both the fluidity properties of liquid and the magnetic properties of solid magnetic materials, which is more helpful for TiO_2_ coating on Fe_3_O_4_ magnetic core.

Many N-containing dyes such Rhodamine B (RhB) could be degraded under visible-light irradiation by a self-photosensitized process, but low photoefficiency and incomplete mineralization make it an urgent issue to develop an efficient visible light photocatalyst for dye photodegradation [25,26]. As a refractory organic dye pollutant, RhB is commonly used as a model to evaluate the activity of photocatalyst. Accordingly, the photocatalytic activity, stability and recoverablility of the prepared Fe_3_O_4_/TiO_2_ nanocomposite were investigated under visible light and solar light by choosing RhB as the target pollutant. The main active species were confirmed and the possible degradation mechanism of RhB was proposed.

## 2. Results and Discussion

### 2.1. Morphologies and Structure Analysis

**Figure 1:**
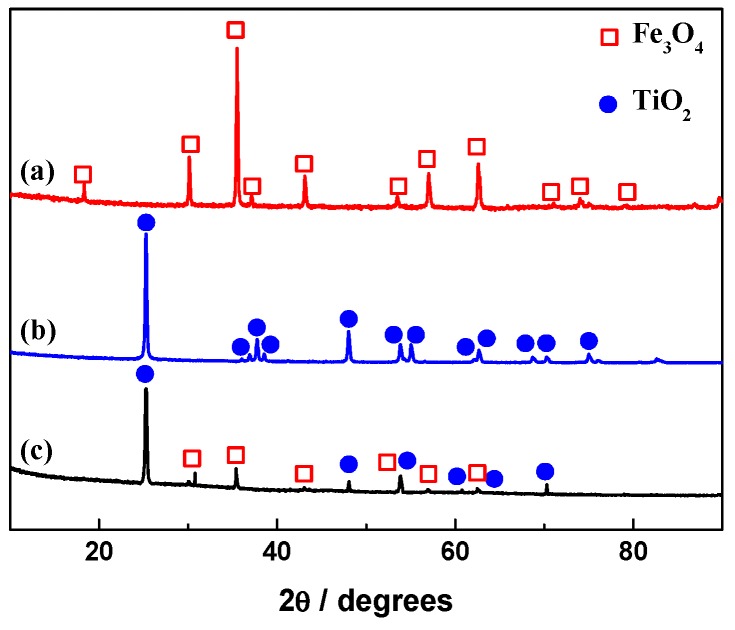
XRD patterns of (**a**) Fe_3_O_4_ magnetic powder; (**b**) TiO_2_ and (**c**) Fe_3_O_4_/TiO_2_ nanocomposite.

Figure 1 shows the XRD spectra of Fe_3_O_4_ magnetic powder, TiO_2_ and the Fe_3_O_4_/TiO_2_ nanocomposite. In Figure 1a, the diffraction peaks at 18.3°, 30.1°, 35.4°, 37.1°, 43.0°, 53.4°, 56.9°, 62.5°, 71.0°, 73.9°and 75.0° can be attributed to those of face-centered cubic Fe_3_O_4_ (111), (220), (311), (222), (400), (422), (511), (440), (620), (533) and (622) crystal faces, respectively, which is consistent with the standard XRD diagram (JCPDS No. 19-0629) for Fe_3_O_4_. The diffraction peaks of Fe_2_O_3_, and other characteristic peaks from impurities were not found, indicating that the iron in the precipitate was converted into Fe_3_O_4_ with good crystallinity. In Figure 1b, the diffraction peaks centered at 25.3°, 36.9°, 37.8°, 38.6°, 48.0°, 53.9°, 55.1°, 62.1°, 62.7°, 68.8°, 70.3° and 75.0° can be indexed to those of anatase type TiO_2_ (101), (103), (004), (112), (200), (105), (211), (213), (204), (116), (220) and (215) crystal faces, respectively, in good agreement with the standard XRD spectrum (JCPDS No. 21-1272) for body-centered tetragonal TiO_2_, which indicates that the product was pure anatase TiO_2_. Compared with XRD patterns of Fe_3_O_4_ and TiO_2_, the diffraction peaks of the Fe_3_O_4_/TiO_2_ nanocomposite in Figure 1c can be indexed to face-centered cubic Fe_3_O_4_ (JCPDS No. 19-0629) and anatase–TiO_2_ (JCPDS No. 21-1272). No other diffraction peaks were observed, indicating that the prepared composite was composed of Fe_3_O_4_ and TiO_2_.

**Figure 2:**
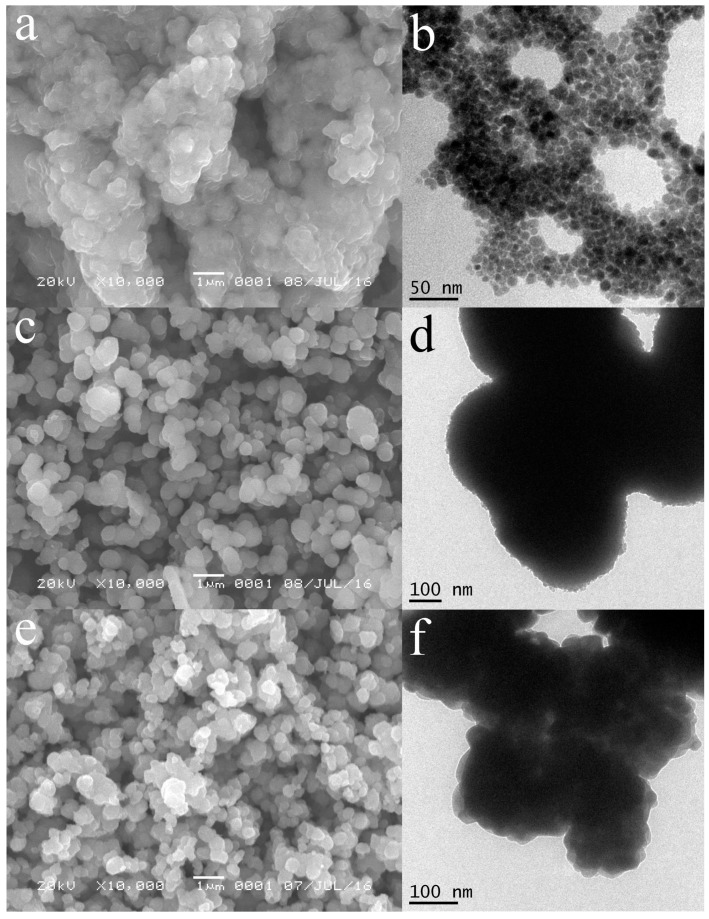
(**a**,**c**,**e**) SEM and (**b**,**d**,**f**) TEM images of (**a**,**b**) Fe_3_O_4_ magnetic powder, (**c**,**d**) TiO_2_, and (**e**,**f**) Fe_3_O_4_/TiO_2_ nanocomposites.

**Scheme 1:**
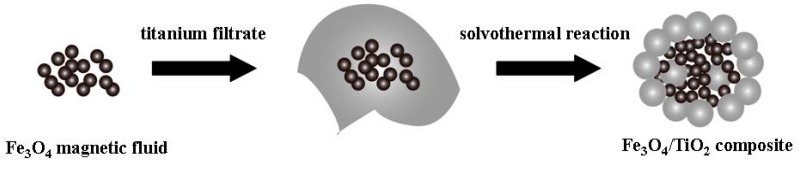
Formation process of Fe_3_O_4_/TiO_2_ nanocomposites.

Figure 2a–f shows the SEM and TEM images of Fe_3_O_4_ magnetic powder, TiO_2_ and the Fe_3_O_4_/TiO_2_ nanocomposite. It can be seen from Figure 2a that the Fe_3_O_4_ magnetic fluid was composed of a large number of spherical nanoparticles with uniform size distribution and a diameter of about 5 nm. Further observation (Figure 2b) shows that the nanoparticles were easy to aggregate with each other, which may be due to the small size and magnetic properties of Fe_3_O_4_ particles. In Figure 2c,d TiO_2_ displays a spherical morphology ranging from 300 nm to 400 nm with poor dispersion and a smooth surface. The SEM image in Figure 2e shows that the nanocomposite was made of a large number of spherical particles with diameters of 100–200 nm and a rough surface. The TEM image in Figure 2f further indicates that the spherical particles were made of nanoparticles with smaller diameters of about 25 nm. Furthermore, the accumulation of the small nanoparticles is helpful to the formation of porous structures. Compared with TiO_2_, the spherical magnetic titania nanocomposite had smaller size and better dispersion, due to the fact that the magnetic fluid inhibited further growth and agglomeration of TiO_2_. The good dispersion of a catalyst is helpful for improving photocatalytic activity. Based on the above analysis, the formation process of Fe_3_O_4_/TiO_2_ nanocomposites is shown in Scheme 1.

The corresponding EDX spectrum in Figure S1 reveals that the Fe_3_O_4_/TiO_2_ nanocomposites mainly contained Ti, Fe and O elements, with a content of 28.72 wt % (Ti element), 23.00 wt % (Fe element) and 48.28 wt % (O element). According to the mass ratio of Fe and Ti, the Fe_3_O_4_ and TiO_2_ contents of the nanocomposite were calculated to be 39.88 wt % and 60.12 wt %, respectively. This is consistent with the addition of titanium filtrate and magnetic fluid in the preparation process, indicating that the Ti and Fe in the precursor were completely converted into TiO_2_ and Fe_3_O_4_ in the magnetic titania nanocomposite.

To further gain insights into the chemical composition, Fe_3_O_4_/TiO_2_ nanocomposition was investigated by XPS, which could verify the valence states of the elements in the material. As depicted in Figure S2a, Ti and Fe could be noticeably observed in the nanocomposite, but the Fe element was not obvious, possible due to the coating of TiO_2_. In Figure S2b the splitting signals at 458.8 eV and 464.4 eV are the characteristic peaks of Ti 2p_3/2_ and Ti 2p_1/2_, indicating that Ti exists in the form of Ti^4+^ in the composite [27]. The Fe 2p XPS spectrum displayed a typical core level spectrum of Fe_3_O_4_ consisting two broad peaks at about 711.2 eV and 724.7 eV (Figure S2c), which correspond to Fe 2p_3/2_ and Fe 2p_1/2_ [28]. The O 1s XPS spectrum in the nanocomposite can be organized into three individual component peaks located at about 529.6 eV, 530.3 eV and 531.2 eV (Figure S2d), which are attributed to lattice oxygen in metal oxide (Fe–O), the O element in the Ti–O bond, and the surface-bound oxygen-containing species, such as OH, which was adsorbed into H_2_O in the surface hydroxyl group (OH), respectively [28,29].

### 2.2. FTIR Analysis

**Figure 3:**
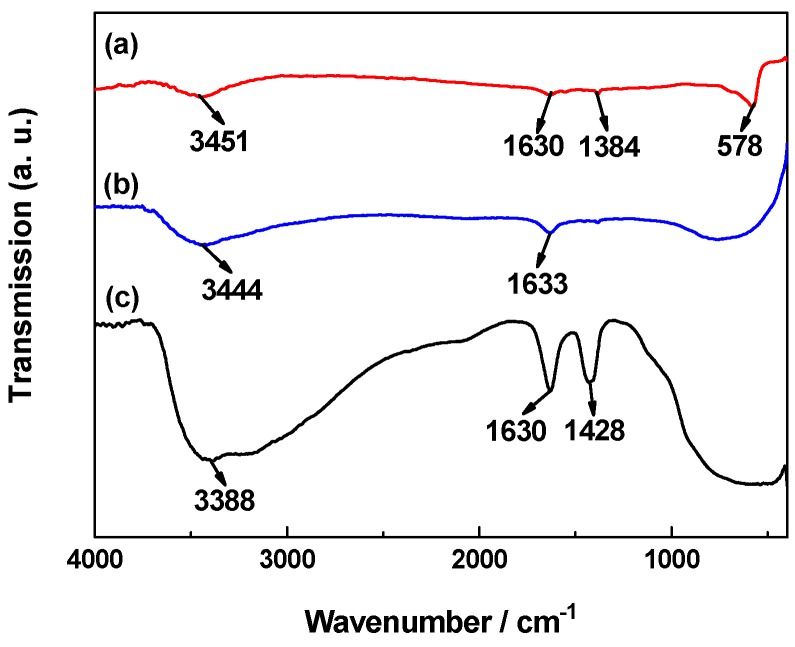
FTIR spectra of (**a**) Fe_3_O_4_ magnetic powder; (**b**) TiO_2_ and (**c**) Fe_3_O_4_/TiO_2_ nanocomposite.

Figure 3 shows the FTIR spectra of Fe_3_O_4_ magnetic powder, TiO_2_ and the Fe_3_O_4_/TiO_2_ nanocomposite. In these spectra, the large and broad absorption peak near 3400 cm^−1^ is related to the stretch region of the surface hydroxyl groups with hydrogen bonds and chemisorbed water [30,31,32]. The absorption peak near 1630 cm^−1^ can be assigned to the O–H bending of molecularly physisorbed water [31,32]. In curve a of Figure 3, the absorption peak at 578 cm^−1^ can be attributed to the formation of the Fe–O bond [33], and the characteristic absorption peak of carbonyl group at 1384 cm^−1^ was derived from the addition of oleic acid during the preparation process of magnetic fluid. In curve b of Figure 3, the large absorption in the range of 600 cm^−1^ to 800 cm^−1^ is characteristic of the formation of an O–Ti–O [30]. In the FTIR spectrum of the Fe_3_O_4_/TiO_2_ nanocomposites, the presence of an absorption peak at the range of 600 cm^−1^ to 800 cm^−1^ and the absence of the characteristic peaks of Fe–O at 578 cm^−1^ indicate that the Fe_3_O_4_ magnetic cores were completely coated with TiO_2_ layer. And the absorption peak at 1428 cm^−1^ corresponding to the –CH=CH– vibration may be due to the incorporation of a small amount of oleic acid in the product. The above results indicate that a small amount of organic molecules were encapsulated into the Fe_3_O_4_/TiO_2_ nanocomposites, which helps to protect the Fe_3_O_4_ magnetic core, eliminates the photodissolution [34] and improves the photocatalytic activity and stability.

### 2.3. UV-Vis Analysis

**Figure 4:**
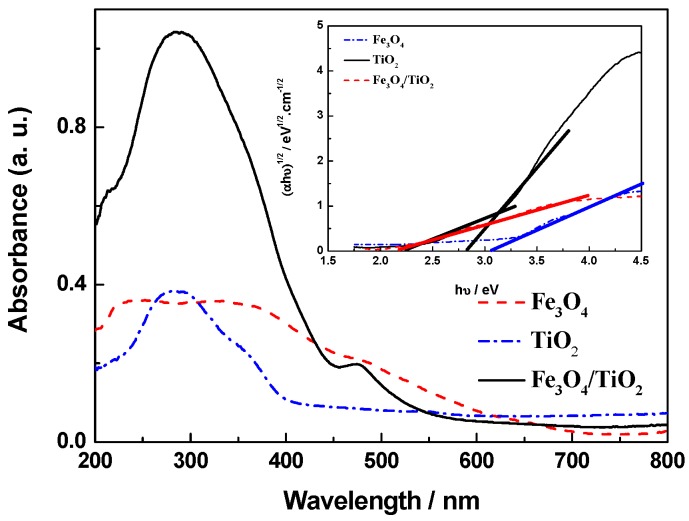
UV-Vis diffusion reflection spectra and plots of (*αhν*)^1/^^2^ versus the photon energy (*hν*) curves (inset) of Fe_3_O_4_ magnetic powder, TiO_2_, and Fe_3_O_4_/TiO_2_ nanocomposite.

The light absorption properties of photocatalysts were investigated by UV-Vis DRS. Figure 4 represents the UV-Vis DRS spectra of Fe_3_O_4_ magnetic powder, TiO_2_ and the Fe_3_O_4_/TiO_2_ nanocomposite. Fe_3_O_4_ magnetic powder had a broad absorption in both ultraviolet and visible light regions, corresponding to the possible photocatalytic activity in these regions. The absorption of TiO_2_ was found in the ultraviolet region of 200–400 nm. Owing to the presence of Fe_3_O_4_ nanoparticles in the sample, the magnetic titania nanocomposite exhibited stronger adsorption than TiO_2_ in the ultraviolet region. Additionally, the adsorption wavelength shifts into the visible light region, indicating that the nanocomposite had a narrower band gap than TiO_2_. As shown in inset of Figure 4, the band gap energy of the samples could be estimated from the linear fit of the linear part of (*αhν*)^1/^^2^ versus (*hν*) plot. These data are also presented in Table 1. A shift in the band gap of magnetic titanic to 2.24 eV for Fe_3_O_4_ and 2.82 eV for TiO_2_, clearly elucidates the presence of interactions and electronic effects between the components due to their contact with each other [35]. The above results suggest that the Fe_3_O_4_/TiO_2_ nanocomposite has strong light absorption properties in the ultraviolet and visible light range and can degrade organic pollutants in the wide spectral range.

### 2.4. Nitrogen Adsorption–Desorption Isotherms

**Figure 5:**
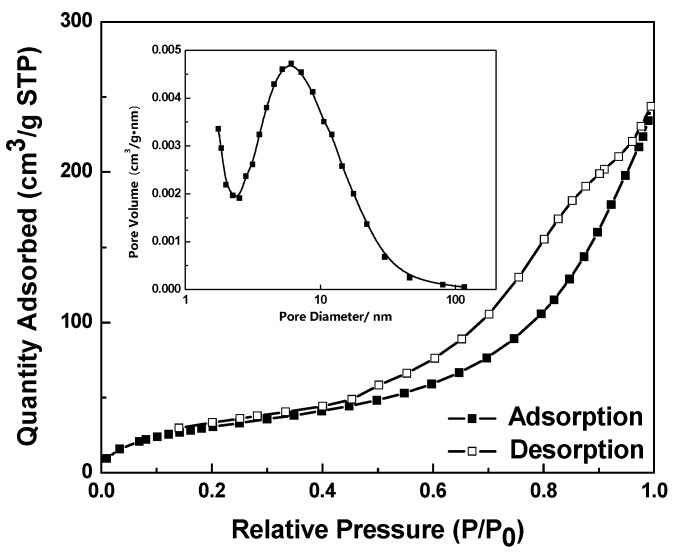
N_2_ adsorption and desorption isotherms and pore size distributions (inset) of Fe_3_O_4_/TiO_2_ nanocomposite.

Figure 5 presents the nitrogen adsorption and desorption isotherms and pore size distribution of the Fe_3_O_4_/TiO_2_ nanocomposite. The BET specific surface area was 119.26 m^2^·g^−1^. As shown in the inset of Figure 5, the pore diameter distribution was measured by the Barret-Joyner-Halenda (BJH) method and the pore size was about 6.1 nm. It may be that the accumulation of a large number of particles with the size of about 25 nm leads to the porous properties. Therefore, the synthesized Fe_3_O_4_/TiO_2_ nanocomposite with porous structures would potentially exhibit excellent photocatalytic properties.

### 2.5. Magnetic Properties

**Figure 6:**
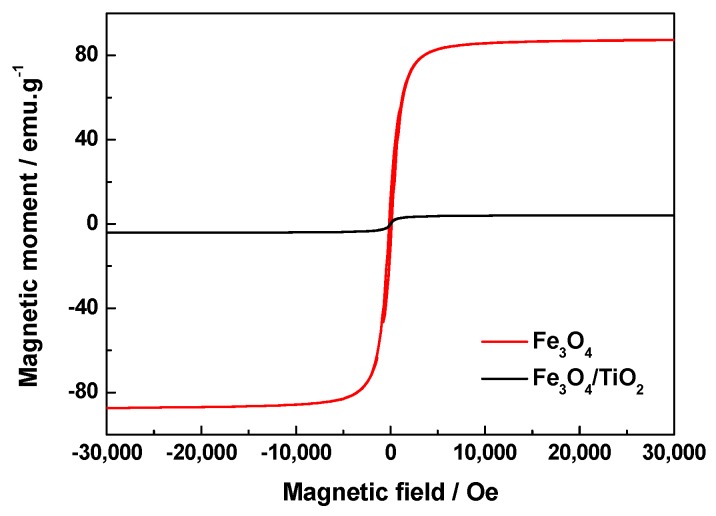
The magnetic hysteresis loops of Fe_3_O_4_ magnetic particles and Fe_3_O_4_/TiO_2_ nanocomposite.

The magnetic measurements of Fe_3_O_4_ magnetic particles and the Fe_3_O_4_/TiO_2_ nanocomposite are displayed in Figure 6. There was no obvious hysteresis for any of the samples, indicating the superparamagnetic behavior at room temperature. The saturation magnetization was measured to be 87.325 emu·g^−1^ for Fe_3_O_4_ magnetic particles and 4.180 emu·g^−1^ for Fe_3_O_4_/TiO_2_ nanocomposite. The TiO_2_ coating has a shielding effect on the magnetic field and hinders the magnetization effect of the Fe_3_O_4_ magnetic core. After the coating of the TiO_2_ shell, the nanocomposite exhibited a low saturation magnetization, but it could still be quickly separated and easily recovered by applying an external magnetic field. The separation and recovery of photocatalysts assisted by magnetic technology is easier to apply than the traditional filtration and centrifugation, exhibiting promising potential in large scale industrial applications.

### 2.6. Photocatalytic Performance

**Figure 7:**
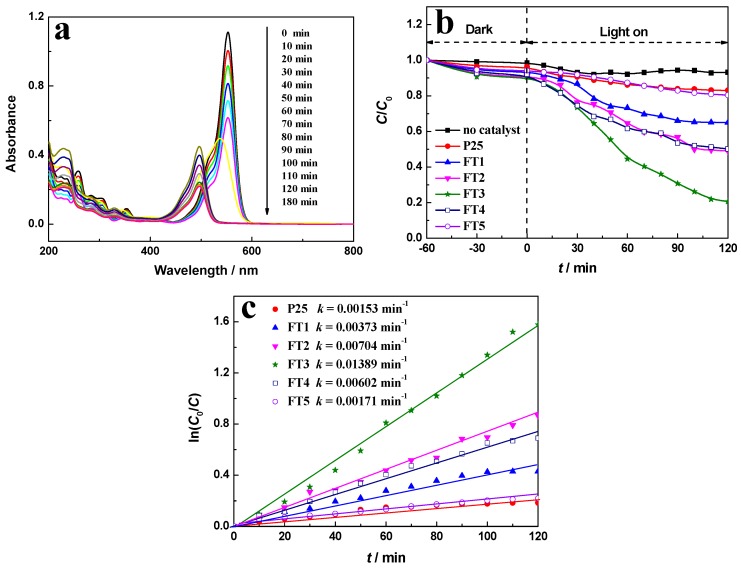
(**a**) Absorption spectra of 5 × 10^−5^ mol/L Rhodamine B (RhB) solution in the presence of FT3 under visible light irradiation at different irradiation times denoted by the different color lines; (**b**) degradation rate of RhB at the different intervals; and (**c**) ln(*C*_0_/*C*) versus *t* curves in the presence of various photocatalysts.

Using the degradation of a 5.0 × 10^−5^ mol·L^−1^ RhB solution as a model reaction and the xenon lamp with a filter (*λ* > 420 nm) as a light source, the photocatalytic performance of the Fe_3_O_4_/TiO_2_ nanocomposite was studied, as shown in Figure 7. In Figure 7a, the maximum adsorption peak of the RhB solution at 554 nm gradually decreases with the increasing irradiation time, indicating the degradation of RhB. After an illumination of 50 min, the maximum absorption peak began to shift and moved to 500 nm in a step-wise manner. The phenomena indicates that more RhB molecules were degraded via the deethylation process in the magnetic titania system [36,37]. When the illumination time exceeded 120 min, the absorbance at about 500 nm hardly changed.

**Figure 8:**
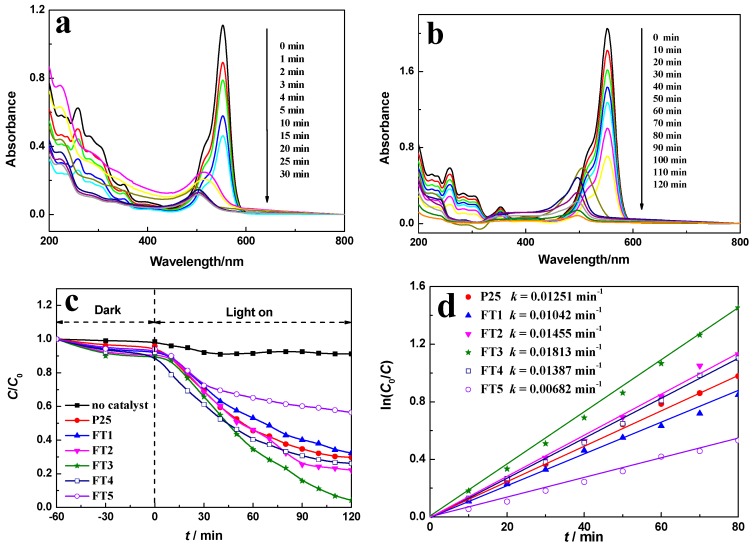
(**a**,**b**) Absorption spectra of (**a**) 5 × 10^−5^ mol·L^−1^ and (**b**) 20 × 10^−5^ mol·L^−1^ RhB solutions in the presence of FT3 under solar light irradiation at different irradiation times denoted by the different color lines; (**c**) degradation rates of RhB solution corresponding to (**b**) at the different intervals; and (**d**) ln(*C*_0_/*C*) versus *t* curves in the presence of various photocatalysts.

Further investigation results as to the effect of Fe_3_O_4_ content in the prepared Fe_3_O_4_/TiO_2_ nanocomposite on the photocatalytic activities are presented in Figure 7b. The Fe_3_O_4_ content in the nanocomposite can be controlled by adjusting the amount of Fe_3_O_4_ magnetic fluid and be determined by EDX, as shown in Table 2. When the addition amount of Fe_3_O_4_ magnetic fluid was 5, 10, 15, 20 and 30 mL, the corresponding products are denoted as FT1, FT2, FT3, FT4 and FT5 with respect to corresponding Fe_3_O_4_ contents of 17.87 wt %, 30.02 wt %, 39.88 wt %, 47.68 wt % and 59.92 wt %, respectively. Before evaluating the catalytic activities of photocatalysts, a blank test was carried out without catalysts, and only 7% of the RhB was photodegradated under the visible light illumination of 120 min. For comparison, the prepared TiO_2_ and P25 as photocatalyst could degrade only 8% and 17% of the RhB, respectively, under the visible light irradiation of 120 min. Although P25 can be excited only by ultraviolet light, the photodegradation rate of RhB with P25 under the visible light irradiation is slightly higher than that of the self-photosensitized process, which may be due to the presence of a small amount of ultraviolet light in the spectrum (Figure S3a). This phenomenon was further confirmed under solar light irradiation as presented in Figure 8. Also, the FT3 nanocomposite could degrade more than 80% of the RhB with an irradiation of 120 min, exhibiting the best photocatalytic activity. The specific surface area of FT3 was 2.6 times higher than that of P25, but the photocatalytic activity of FT3 was much higher than that of P25, indicating that the high photocatalytic activity wasn’t determined only by a large specific surface area. The degradation of RhB was obviously enhanced in the presence of Fe_3_O_4_/TiO_2_ nanocomposites, indicating that the presence of Fe_3_O_4_ in the sample can reduce the band gap and enhance visible light photocatalytic activity. Furthermore, the presence of organic molecules in Fe_3_O_4_/TiO_2_ nanocomposites can inhibit the photodissolution [38], leading to high photocatalytic activity of Fe_3_O_4_/TiO_2_ nanocomposites. The possible reason for this phenomenon is that with the increase of Fe_3_O_4_ content in the composite, TiO_2_ can’t completely envelope the Fe_3_O_4_ inside. Under light irradiation, the strong photocatalytic reaction of TiO_2_ erodes the inert insulating organic layer, and the Fe_3_O_4_ magnetic core then has direct contact with TiO_2_, which leads to the occurrence of photodissolution [38] and the decrease of photocatalytic activity.

Using the linear simulation of RhB concentration, the RhB degradation kinetic was also investigated by a pseudo first-order model called the Langmuir–Hinshelwood (L–H) model [39,40,41]. The L–H model is well established for heterogenerous photocatalysis at low dye concentration. The kinetic equation (Equation (1)) is shown as follows:

*r* = −d*C*/d*t* = *K*_app_*C* (1)

where *r* is the degradation rate, *C* is the concentration of RhB at the various intervals of time, and *K*_app_ represents the apparent first-order rate constant (min^−1^). By integrating Equation (1), we can obtain the linear plot of ln(*C*_0_/*C*) versus time (*t*) as follows (Equation (2)):

ln (*C*_0_/*C*) = *K*_app_*t* (2)

where *C*_0_ is the initial concentration of RhB and *K*_app_ can be calculated from the gradient of the curve. It is clear that good linear relationships can be obtained for the experimental data. Based on the calculation, the *K*_app_ values were estimated and are shown in the inset of Figure 7c. The Fe_3_O_4_/TiO_2_ nanocomposites exhibited higher *K*_app_ values than P25. Among them, the FT3 nanocomposite showed the largest *K*_app_ value, of about 9.08 times larger than that of P25, which is consistent with Figure 7b.

In similar experimental conditions, the photocatalytic behavior of Fe_3_O_4_/TiO_2_ nanocomposites under solar light was further investigated, as presented in Figure 8. As shown in Figure 8a, at the irradiation time of only 30 min, more than 95% of the RhB was degraded, indicating a rapid and efficient photocatalytic reaction assisted by the magnetic titania nanocomposite. With the high concentration of 20 × 10^−5^ mol·L^−1^ RhB solution, the degradation rate reached 95% under solar light irradiation for 120 min, which is obviously superior to that which occurred under visible light illumination. Corresponding to Figure 8b, the effect of the Fe_3_O_4_ amount in Fe_3_O_4_/TiO_2_ nanocomposites on the photocatalytic behavior of the RhB solution is displayed in Figure 8c. All the photocatalysts had an effect on the photocatalytic degradation of RhB under solar light. FT3 exhibited the highest photocatalytic activity, which was similar to that shown in Figure 7.

To gain direct evidence of the photocatalytic activity, the PL spectra were carried out to investigate the recombination rate of photogenerated electrons and holes. The energy released by the electron–hole recombination is in direct proportion to the recombination rate and inversely proportional to the photocatlytic activity. As shown in Figure S4, the synthesized TiO_2_ exhibited a stronger PL intensity than the Fe_3_O_4_/TiO_2_ nanocomposites did. It can be observed that the PL intensity of the FT3 nanocomposite was considerably less than that of other Fe_3_O_4_/TiO_2_ nanocomposites, suggesting that the FT3 nanocomposite had the lowest recombination rate of electrons and holes under light irradiation. Therefore, the appropriate Fe_3_O_4_ quantity (39.88 wt %) in the composite is very important for enhancing the photocatalytic activity.

### 2.7. Proposed Photocatalytic Degradation Mechanism

**Figure 9:**
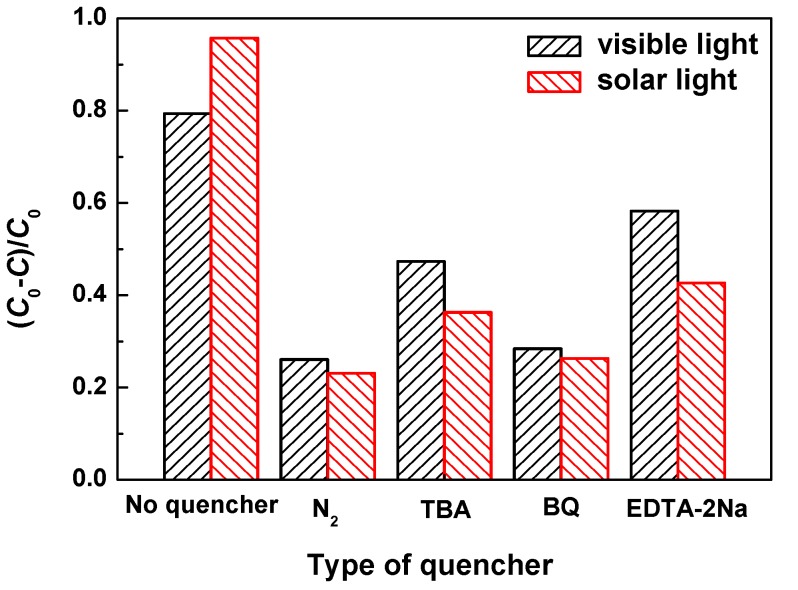
Effects of different quenchers on photocatalytic degradation of RhB with FT3.

**Scheme 2:**
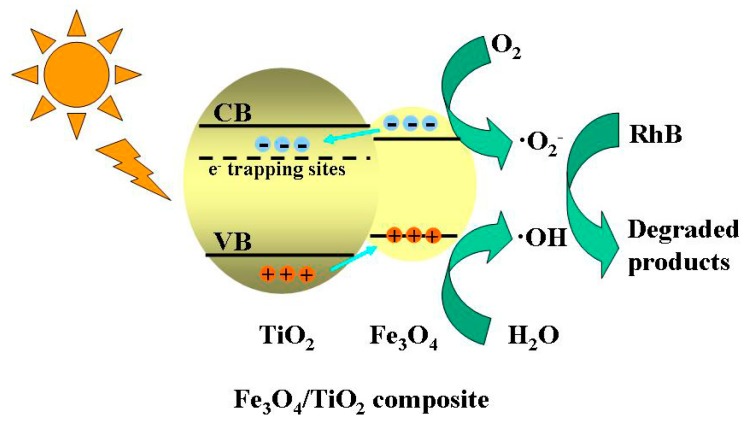
The schematic diagram of the proposed photocatalytic mechanism of RhB for Fe_3_O_4_/TiO_2_ nanocomposite.

During the photocatalytic degradation of organic pollutants, the hydroxyl radical (·OH), superoxide radical (·O_2_^−^) and hole (h^+^) are generally accepted as the main active species [42]. Thus the trapping experiments of active species were carried out to make clear the possible photocatalytic mechanism over the FT3 nanocomposite. Different scavengers were used to consume the corresponding active species on the photocatalytic degradation of RhB. Tert-butyl alcohol (TBA), benzoquinone (BQ) and disodium ethylenediaminetetraacetate dehydrate (EDTA-2Na) were employed as scavengers of ·OH, ·O_2_^−^ and h^+^, respectively. The photocatalytic degradation rates of RhB with FT3 under visible light and solar light irradiation of 120 min in the presence of different quenchers are shown in Figure 9. The effects of a series of quenchers on the degradation rates of RhB under solar light irradiation were similar with that under visible light illumination. In comparison to the blank experiment with no quencher, BQ showed the most significant effect on the RhB degradation, indicating that ·O_2_^−^ was the main active species. At the same time both TBA and EDTA–2Na decreased the degradation rates of RhB to some extent, suggesting that ·OH and h^+^ also play an important role in the photocatalytic process. Besides, the photocatalytic reaction of RhB with FT3 was performed under an N_2_ atmosphere. The degradation rate of RhB decreased dramatically, further confirming the importance of the ·O_2_^−^ active species generated from the O_2_ molecules. Combined with the band gap of the nanocomposite, the possible photocatalytic mechanism of the Fe_3_O_4_/TiO_2_ composite is described in Scheme 2 [17].

### 2.8. Magnetic Recyclability

**Figure 10:**
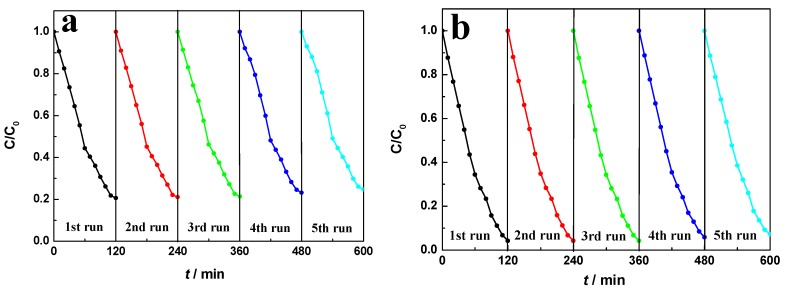
Recycle curves of FT3 for the degradation of the (**a**) 5 × 10^−5^ mol·L^−1^ RhB solution under visible light irradiation and the (**b**) 2 × 10^−5^ mol·L^−1^ RhB solution under solar light irradiation.

The stability of the catalytic materials is of great importance if the catalysts are to be practically applicable. The prepared Fe_3_O_4_/TiO_2_ nanocomposite can be easily separated for reuse from the RhB solution by the addition of an external magnetic field. Figure 10 shows the recycling test of the FT3 nanocomposite for RhB degradation under both visible light and solar light. After five recycles of the photocatalytic degradation of RhB, the catalytic activities of the FT3 composite showed just a slight decrease. As shown in Figure 10, the degradation rates of the RhB solution were 75.3% for visible light and 92.8% for solar light, which can reach up to 95% and 97% of the first RhB degradation rate, respectively. The above results suggest that Fe_3_O_4_/TiO_2_ nanocomposite has good cyclic stability under visible light and solar light, and that the stability under solar light is superior to that under visible light. As a magnetic photocatalyst, magnetic field can effectively achieve the separation and recovery of the catalyst.

### 2.9. Photocurrent Responses

**Figure 11:**
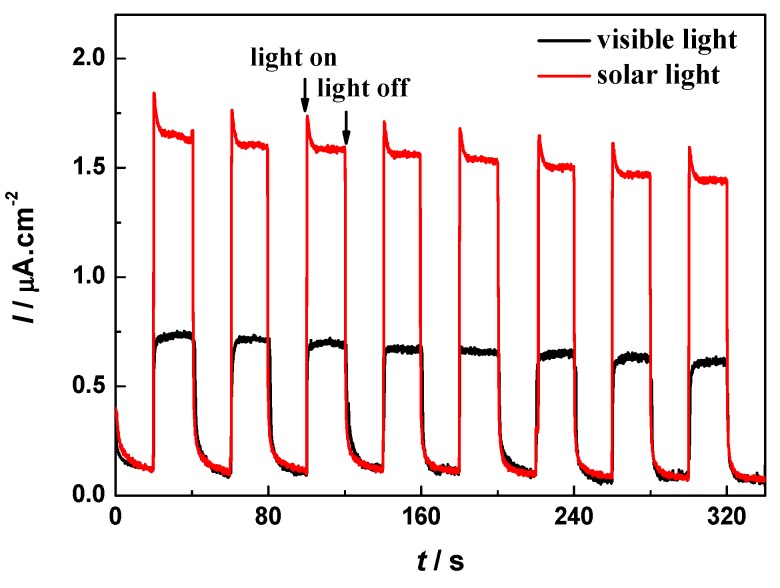
Transient photocurrent responses for FT3 under both visible light and solar light.

To further investigate the charge-transfer properties at the semiconductor interfaces, transient photocurrent responses of the FT3 nanocomposite are recorded in Figure 11. It was observed that the photocurrents could reproducibly increase under each visible light and solar light irradiation, and quickly recover in dark. Also, the FT3 nanocomposite exhibited a maximum photocurrent density of 1.62 μA·cm^−2^ under solar light illumination, which is about 2.3 times as much as that under visible light irradiation. The high photocurrent of the FT3 nanocomposite suggests a low recombination rate of electrons and holes under solar light irradiation.

## 3. Materials and Methods

### 3.1. Materials

Ilmenite (99%) was purchased from Consolidated Rutile Ltd. (Murarrie, Australia), and its chemical composition is TiO_2_ 10.76 wt %, FeTiO_3_ 76.25 wt %, Al_2_O_3_ 3.14 wt % and SiO_2_ 9.85 wt %. Degussa P25 titanium dioxide (Evonik, Degussa, Frankfurt, Germany) has a specific surface area of 46 m^2^·g^−1^ and contains 80% anatase and 20% rutile. Other chemical regents were of analytical grade and were purchased from J&K Chemical Ltd. (Beijing, China). They were used as received without any purification. Deionized water was used throughout the experiments.

### 3.2. Synthesis

About 20 g of ilmenite was put into a ball milling jar together with 4 stainless steel balls of a diameter of 25.4 mm. After ball milling for 3 h at a speed of 200 r·min^−1^, the ilmenite powder was soaked in 60 mL of concentrated sulfuric acid at 150 °C for 6 h. After cooled to ambient temperature, the mixture was diluted by adding distilled water, stirred continuously, and filtered to obtain a reddish brown leaching solution. 5 mol·L^−1^ NaOH solution was added to the above leaching solution until the brown product precipitated completely. The iron precipitation was separated from titanium filtrate by suction filtration and the obtained filtrate contained 0.16 mol·L^−1^ titanium. The iron precipitate was washed by deionized water, and dried to obtain the ferric hydroxide solid. An amount of 5.0 g of the ferric hydroxide solid was added to 40 mL of hydrazine hydrate, 140 mL of deionized water, 1 mmol of polyethylene glycol 800 and 5 mL of oleic acid in turn. After ultrasonic vibration, the above mixture was heated to 120 °C for 3 h. The filtrate obtained by suction filtration was washed repeatedly by ethanol and deionized water. The Fe_3_O_4_ magnetic fluid with 0.10 mol·L^−1^ Fe_3_O_4_ was further dried under vacuum at 80 °C for 12 h. The obtained magnetic powder can be used for characterization.

The pH value of 40 mL titanium filtrate was adjusted to 7 by adding 50% sulfuric acid solution. After that, 1 mL concentrated ammonia solution and 15 mL Fe_3_O_4_ magnetic fluid were added to the above solution, and then it was sealed in a Teflon-lined stainless-steel autoclave (100 mL capacity). The autoclave was placed in an oven at 140 °C for 6 h. The prepared product was collected by centrifugation, rinsed with ethanol and deionized water, and dried at 80 °C for 12 h. The product was the Fe_3_O_4_/TiO_2_ nanocomposites. Using the same method, TiO_2_ can be prepared without the addition of the Fe_3_O_4_ magnetic fluid. In the controlled experiments, Degussa P25 was used to evaluate the prepared photocatalysts under the same conditions for comparison.

### 3.3. Characterization

The morphologies and nanostructures of the samples were revealed with a JEOL JSM-6360LV scanning electron microscopy (SEM) (JEOL Ltd., Tokyo, Japan) and a JEM-1011 transmission electron microscopy (TEM) (JEOL Ltd., Tokyo, Japan). The energy-dispersive X-ray (EDX) analysis was performed on the SEM microscope equipped with an Oxford X-act EDX analyzer (Oxford Instruments, Oxford, UK). X-ray powder diffraction (XRD) was obtained on the Empyrean XRD-6000 (PANalytical, Almelo, Holland) using a Cu Kα radiation source (λ = 0.15406 nm). Fourier transform infrared (FTIR) measurements were performed on a Nicolet NEXUS spectrometer (Nicolet Corp., Ltd., Madison, WI, USA) using KBr pellets. The Brunauer-Emmett-Teller (BET) surface area and pore volume of the sample were measured by nitrogen adsorption on the Empyrean apparatus (Micromeriticas Corp., Norcross, GA, USA). UV-Vis diffusion reflection spectra (UV-Vis DRS) were recorded on a UV-2550 spectrometer (Shimadzu, Kyoto, Japan) with BaSO_4_ as a reference. X-ray photoelectron spectroscopy (XPS) analysis was performed on a Physical Electronics PHI 1600 ESCA system (Physical Electronics Inc., Chanhassen, MN, USA) with Al K*α* radiation and a multichannel detector, and all the binding energies were referenced to the C 1s peak at 284.6 eV. Magnetic hysteresis curves were evaluated using a vibrating sample magnetometer (VSM attachment on PPMS Dynacool System, Quantum Design, San Diego, CA, USA) at room temperature. Photocurrent measurements were performed in a three-electrode system by a CHI 760E electrochemical workstation (Chenhua, Shanghai, China). The photocurrent curves were recorded at 0 V (vs. Ag/AgCl) in 0.5 mol·L^−1^ of Na_2_SO_4_ solution with a 250-W xenon arc lamp with a UV cutoff glass filter (λ > 420 nm) and an AM 1.5 G filter. The photoluminescence (PL) emission spectra were recorded on an F-7000 FL spectrophotometer (Hitachi Ltd., Tokyo, Japan) at room temperature.

### 3.4. Photocatalytic Activity

Photocatalytic activities of the catalysts were evaluated by the degradation of RhB at ambient temperatures. In a typical reaction, 20 mg of photocatalyst was dispersed into 100 mL of RhB solution in a quartz photoreactor . Prior to the light irradiation, the above suspension was stirred in the dark for 60 min in order to reach the adsorption/desorption equilibrium of dye molecules on the photocatalysts. A 250-W xenon arc lamp with a UV cutoff filter (λ > 420 nm, about 100 mW·cm^−2^) was used as the visible light source, and with an AM 1.5G filter (about 100 mW·cm^−2^) was employed as the solar light source. The reactor was placed 10–15 cm away from the light source in order to adjust the light to the same intensity. The irradiance spectra of both visible light and solar light are listed in Figure S3. At certain time intervals, 3 mL of the suspension was withdrawn and filtered through a 0.22 μm membrane filter made of polytetrafluoroethylene (PTFE) to obtain a clear solution. The RhB concentration was determined by the characteristic adsorption intensity at 554 nm as a monitored parameter using a UV-Vis spectrometer.

### 3.5. Trapping Experiments of Active Species

To investigate the active species involved in the photocatalytic reaction, a series of quenching tests were carried out by the addition of a scavenger to the reaction system. Tert-butyl alcohol (TBA, 1 mmol·L^−1^), benzoquinone (BQ, 1 mmol·L^−1^) and disodium ethylenediaminetetraacetate dehydrate (EDTA-2Na, 1 mmol·L^−1^) were selected as the scavengers of hydroxide radicals (·OH), superoxide radicals (·O_2_^−^) and photogenerated holes (h^+^), respectively.

## 4. Conclusions

Using mineral-rich and inexpensive ilmenite as a source of titanium and iron, Fe_3_O_4_ magnetic fluid and titanium filtrate were effectively prepared and mixed, resulting in the preparation of a magnetic titania nanocomposite at low cost. The as-prepared product was constructed with face-centered cubic Fe_3_O_4_ and anatase TiO_2_, and had a porous structure and strong adsorption for visible light. A small amount of organic molecules encapsulated into the magnetic composite eliminated the occurrence of photodissolution and improved the catalytic stability. The photocatalytic activity and cycling stability of Fe_3_O_4_/TiO_2_ nanocomposites under both visible light and solar light were evaluated by the degradation reaction of an RhB solution. Fe_3_O_4_/TiO_2_ nanocomposites with a Fe_3_O_4_ content of 39.88 wt % exhibited the best photocatalytic activity for the degradation of the RhB solution. The recovered Fe_3_O_4_/TiO_2_ nanocomposites by magnetic field separation displayed excellent photocatalytic activity, suggesting high photocatalytic stability and a potential application in wastewater treatment.

## Figures and Tables

**Table 1:** The band gap of Fe_3_O_4_ magnetic powder, TiO_2_ and Fe_3_O_4_/TiO_2_ nanocomposite.

Samples	Band Gap/eV
Fe_3_O_4_ magnetic powder	2.17
TiO_2_	3.07
Fe_3_O_4_/TiO_2_ nanocomposite	2.24 (for Fe_3_O_4_) 2.82 (for TiO_2_)

**Table 2:** Fe_3_O_4_ and TiO_2_ contents of the as-prepared Fe_3_O_4_/TiO_2_ nanocomposites detected by energy-dispersive X-ray (EDX).

Samples	Fe_3_O_4_ Magnetic Fluid/mL	Fe_3_O_4_ Content/wt %	TiO_2_ Content/wt %
FT1	5	17.87	82.19
FT2	10	30.02	69.98
FT3	15	39.88	60.12
FT4	20	47.68	52.32
FT5	30	59.92	40.08

## Supplementary Materials

Supplementary materials are available online. Figure S1: EDX pattern of Fe_3_O_4_/TiO_2_ nanocomposite, Figure S2: The wide spectrum (**a**), Ti 2p (**b**), Fe 2p (**c**) and O1s (**d**) XPS spectra of Fe_3_O_4_/TiO_2_ nanocomposite, Figure S3: The irradiance spectra of 250-W xenon arc lamp with a UV cutoff filter (λ > 420 nm) (a) and with an AM 1.5G filter (b), Figure S4: PL spectra for P25, FT1, FT2, FT3, FT4 and FT5.
