# Supplementary material for: Recyclable Magnetic Titania Nanocomposite from Ilmenite with Enhanced Photocatalytic Activity
Source: Molecules. 2017 Nov 23;22(12):2044. doi: 10.3390/molecules22122044 (PMC6149852; doi:10.3390/molecules22122044)
Supplement: Supplementary file 1 [file molecules-22-02044-s001.pdf]

Recyclable Magnetic Titania Nanocomposite from Ilmenite with Enhanced Photocatalytic Activity

Tianjie Hong[1], Jun Mao[1], Feifei Tao[1,2,*] and Mingxuan Lan[1]

1 Department of Chemistry and Chemical Engineering, Shaoxing Univeristy, Shaoxing 312000, P. R. China

hongtianjie001@163.com (T.H.); maojune666@163.com (J. M.); 13467181280@163.com (M.L.)

[2] Shanghai Advanced Research Institute, Chinese Academy of Sciences, Shanghai 201210, P. R. China

[*] Correspondence: feifeitao@usx.edu.cn; Tel.: +86-575-8834-2505.

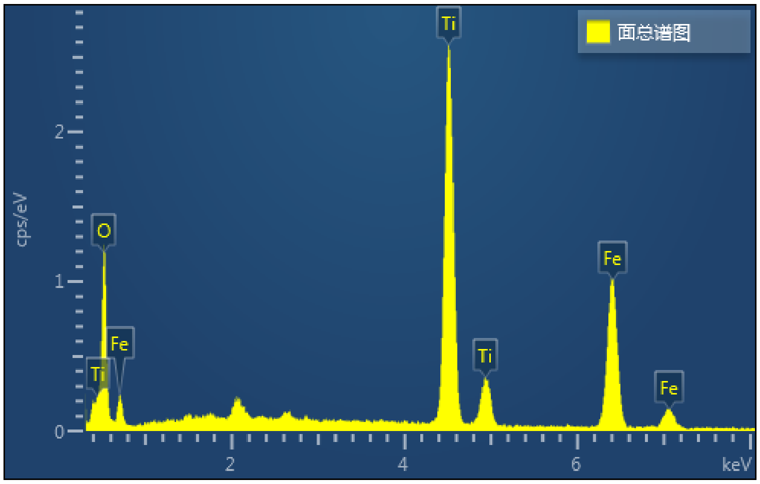

Figure S1. EDX pattern of Fe_3O_4/TiO_2 nanocomposite.

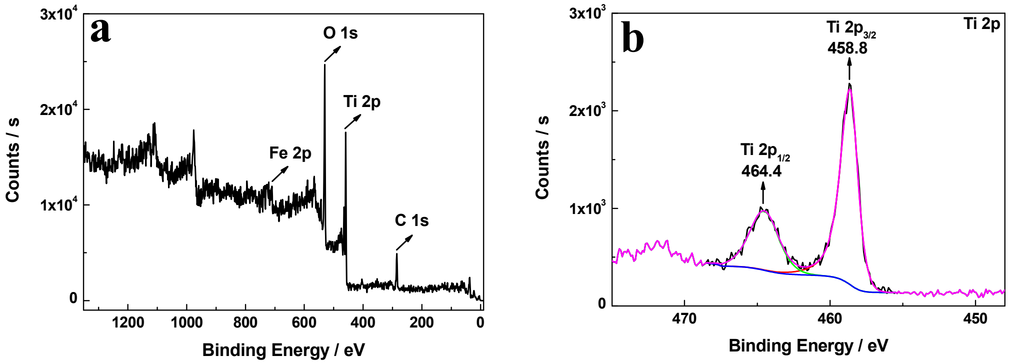

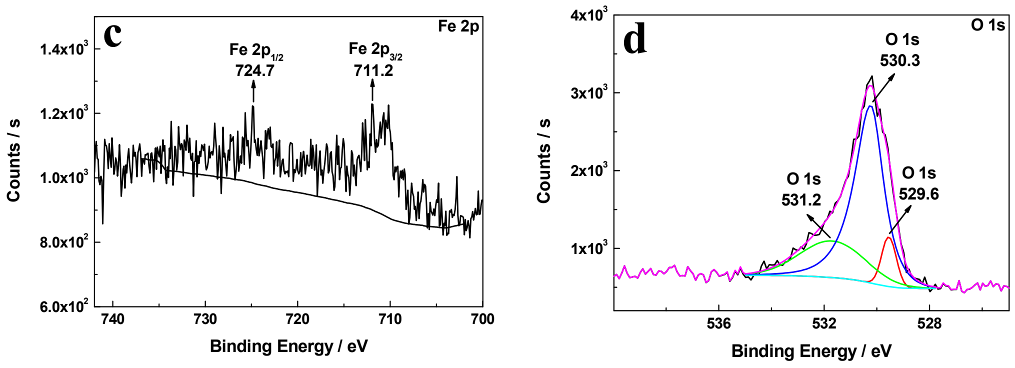

Figure S2. The wide spectrum (a), Ti 2p (b), Fe 2p (c) and O1s (d) XPS spectra of Fe_3O_4/TiO_2 nanocomposite.

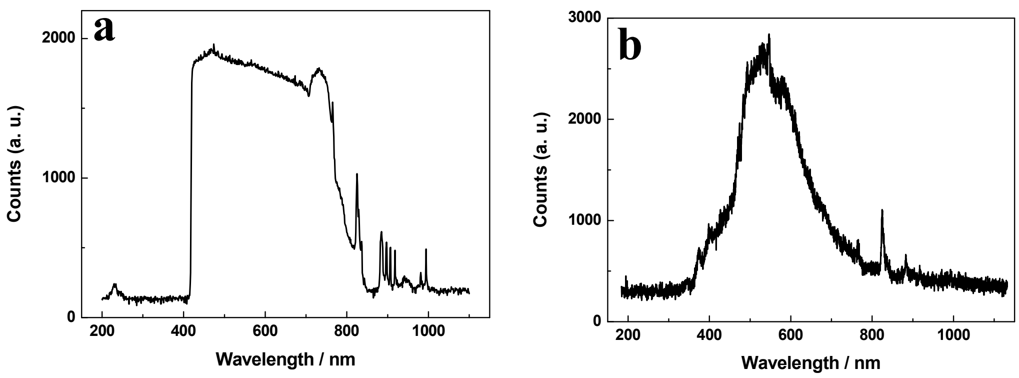

Figure S3. The irradiance spectra of 250-W xenon arc lamp with a UV cutoff filter (λ>420 nm) (a) and with an AM 1.5G filter (b).

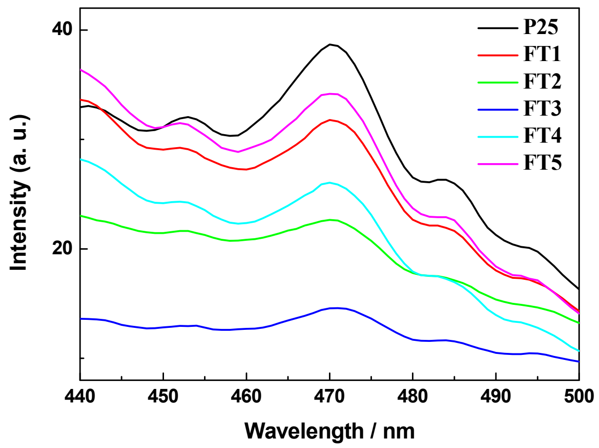

Figure S4. PL spectra for P25, FT1, FT2, FT3, FT4 and FT5.
